# The arachidonic acid metabolite 11,12-epoxyeicosatrienoic acid alleviates pulmonary fibrosis

**DOI:** 10.1038/s12276-021-00618-7

**Published:** 2021-05-14

**Authors:** Hak Su Kim, Su-Jin Moon, Sang Eun Lee, Gi Won Hwang, Hyun Ju Yoo, Jin Woo Song

**Affiliations:** 1 Department of Pulmonary and Critical Care Medicine, Asan Medical Institute of Convergence Science and Technology, Asan Medical Center, University of Ulsan College of Medicine, Seoul, Republic of Korea; 2 Veterans Medical Research Institute, Veterans Health Service Medical Center, Seoul, Republic of Korea; 3 Department of Convergence Medicine, Asan Medical Institute of Convergence Science and Technology, Asan Medical Center, University of Ulsan College of Medicine, Seoul, Republic of Korea

**Keywords:** Respiratory tract diseases, Chronic inflammation

## Abstract

Epoxyeicosatrienoic acids (EETs) are metabolites of arachidonic acid that are rapidly metabolized into diols by soluble epoxide hydrolase (sEH). sEH inhibition has been shown to increase the biological activity of EETs, which are known to have anti-inflammatory properties. However, the role of EETs in pulmonary fibrosis remains unexplored. Liquid chromatography with tandem mass spectrometry (LC-MS/MS) was used to analyze EETs in the lung tissues of patients with idiopathic pulmonary fibrosis (IPF, *n* = 29) and controls (*n* = 15), and the function of 11,12-EET was evaluated in in vitro and in vivo in pulmonary fibrosis models. EET levels in IPF lung tissues, including those of 8,9-EET, 11,12-EET, and 14,15-EET, were significantly lower than those in control tissues. The 11,12-EET/11,12-DHET ratio in human lung tissues also differentiated IPF from control tissues. 11,12-EET significantly decreased transforming growth factor (TGF)-β1-induced expression of α-smooth muscle actin (SMA) and collagen type-I in MRC-5 cells and primary fibroblasts from IPF patients. sEH-specific siRNA and 1-trifluoromethoxyphenyl-3-(1-propionylpiperidin-4-yl) urea (TPPU; sEH inhibitor) also decreased TGF-β1-induced expression of α-SMA and collagen type-I in fibroblasts. Moreover, 11,12-EET and TPPU decreased TGF-β1-induced p-Smad2/3 and extracellular-signal-regulated kinase (ERK) expression in primary fibroblasts from patients with IPF and fibronectin expression in Beas-2B cells. TPPU decreased the levels of hydroxyproline in the lungs of bleomycin-induced mice. 11,12-EET or sEH inhibitors could inhibit pulmonary fibrosis by regulating TGF-β1-induced profibrotic signaling, suggesting that 11,12-EET and the regulation of EETs could serve as potential therapeutic targets for IPF treatment.

## Pulmonary fibrosis: Suppressing creation of scar tissue

Signaling molecules called eicosanoids, which are derived from fatty acids, can suppress lung damage in idiopathic pulmonary fibrosis (IPF), a chronic, progressive disease in which scar tissue builds up in the lungs, making it hard to breathe. The causes of IPF are unknown. Eicosanoids, which have anti-inflammatory properties, have been studied in various lung diseases. Jin Woo Song at the University of Ulsan College of Medicine in Seoul, South Korea, and co-workers investigated how they might affect IPF. They found that eicosanoid levels were lower in lung tissues from patients with IPF than in healthy tissues. Further investigation showed eicosanoid levels could be boosted by suppressing an enzyme called sEH that degrades them. Thus, suppression of sEH and boosting of eicosanoid levels show promise as therapeutic targets for IPF.

## Introduction

Idiopathic pulmonary fibrosis (IPF) is a chronic progressive fibrosing interstitial pneumonia of unknown etiology characterized by irreversible loss of lung function and poor prognosis^1^. Aberrant wound healing responses to lung injury have been implicated in the pathogenesis of pulmonary fibrosis^2^. The characteristic progressive fibrosis in IPF is due to fibroblast activation, which is regulated by various metabolic mediators, including lipid metabolites^3–5^. Previous studies have suggested that metabolic dysregulation, such as reduced sphingolipid metabolism, altered fatty acid oxidation, and an increased number of dysfunctional mitochondria, contributes to the pathogenesis of IPF^6–8^. However, the metabolic mediators associated with the pathogenesis of IPF have not been fully identified.

Eicosanoid metabolites of arachidonic acid play various roles in disease pathogenesis, such as anti-inflammatory, antifibrogenic and antiapoptotic roles^3,5,9,10^. Some eicosanoids, including prostaglandin E2, lipoxin A4, and prostacyclin, have shown antifibrotic effects on fibrosis models^3,11,12^. Epoxyeicosatrienoic acids (EETs), including 5,6-EET, 8,9-EET, 11,12-EET, and 14,15-EET, are biologically active eicosanoids that are produced by the actions of cytochrome P450 (CYP) epoxygenases on arachidonic acid. EETs are rapidly metabolized by soluble epoxide hydrolase (sEH) into dihydroxyeicosatrienoic acids (DHETs), which are the less active diol forms, and inhibiting sEH activity increases the levels of EETs in tissues and plasma^13^. Previous studies have suggested that epoxide hydrolase and epoxygenase metabolites can serve as key mediators during the pathogenesis of fibrosis and as therapeutic targets^14–18^. In the lung, EETs are the dominant eicosanoids produced in response to microbial challenge^19^ and are involved in the development of many pulmonary diseases, including asthma^20^, chronic obstructive pulmonary disease^21^, and pulmonary fibrosis^17^. In a nephrectomized rat model, EETs inhibited α-SMA expression and TGF-β1-Smad signaling and protected against renal fibrosis^14^. Moreover, inhibiting sEH prevents renal^15^, liver^16^, heart^22^, and pulmonary fibrosis^17^. However, the compositional changes in EETs in the lung tissues of patients with IPF and the roles of EETs in the pathogenesis of IPF have not been completely explored.

In this study, we hypothesized that patients with IPF exhibited changes in eicosanoid composition, which would affect IPF pathogenesis. We performed eicosanoid profiling in human lung tissues using LC-MS/MS and found that the levels of EETs, especially 11,12-EET, were reduced in the lung tissues of patients with IPF compared to those in control subjects. The role of 11,12-EET was investigated using in vitro and in vivo pulmonary fibrosis models.

## Materials and methods

### Human subjects

All patients with IPF met the diagnostic criteria set by the American Thoracic Society/European Respiratory Society/Japanese Respiratory Society/Latin American Thoracic Association^23^. IPF lung tissues (*n* = 29) were obtained at the time of surgical lung biopsy, and control human lung tissues (*n* = 15) with no histological evidence of disease were obtained from the Bio-Resource Center of Asan Medical Center, Seoul, Republic of Korea.

### Ethics statement

Lung tissue procurement was completed under Protocol #2016-1131, which was approved by the Institutional Review Board of Asan Medical Center, Seoul, Republic of Korea. Informed written consent was obtained from all study participants. Mouse experiments were performed in accordance with the Guiding Principles for the Care and Use of Animals, and protocols were approved by the Animal Care and Handling Committee of Asan Medical Center (protocol #2017-12-034). While conducting the experiments, care was taken so that animal suffering was minimized.

### LC-MS/MS analysis of eicosanoids

Eicosanoids were extracted from human lung tissues (~20 mg) using solid phase extraction (SPE). Eicosanoids were eluted with 0.5 mL of methanol followed by 1.5 mL of ethyl acetate and analyzed by a liquid chromatography-tandem mass spectrometry system. Detailed protocols for the analysis of eicosanoids can be found in the online supplement.

### Animal model

Six-week-old female C57BL/6J mice were obtained from Orient Bio (Seongnam, South Korea) and acclimatized for 2 weeks before the experiments. The mice were housed under specific pathogen-free conditions in an air conditioned (22 ± 2 °C) and humidity-controlled (45–55%) room under a 12-h light and 12-h dark cycle with ad libitum access to food and water. The mice were randomly divided into four groups and were administered (1) saline plus vehicle, (2) saline plus TPPU, (3) bleomycin (3 U/kg) plus vehicle, or (4) bleomycin plus TPPU. The mice were anesthetized with intraperitoneal injections of 50 mg/kg alfaxalone (Jurox, Rutherford, NSW, Australia) and 5 mg/kg xylazine (Bayer, Leverkusen, Germany). Saline or bleomycin in saline was injected intratracheally. After bleomycin administration, 0.25 mg/kg TPPU was administered immediately by intraperitoneal injection (5 days per week for 3 weeks). The mice were euthanized, and the lungs were harvested on day 21, snap frozen in liquid nitrogen, and stored at −80 °C.

### Hydroxyproline assay

To estimate the amount of collagen in lung tissues, a hydroxyproline assay was performed using a commercial kit (BioVision, Milpitas, CA, USA) according to the manufacturer’s protocol. The lungs were weighed, homogenized in sterile water, and hydrolyzed in 12 N HCl at 120 °C for 3 h. The hydrolyzed samples were incubated with 4‐(dimethylamino) benzaldehyde for 90 min at 60 °C, and the absorbance of the oxidized hydroxyproline was measured at 560 nm. The amount of hydroxyproline is expressed as μg/mg of lung tissue.

### Cell culture

Beas-2B cells, a human bronchial epithelial cell line (ATCC, Manassas, VA, USA), were cultured in bronchial epithelial cell culture medium (Lonza, Bend, OR, USA) at 37 °C in a humidified atmosphere containing 5% CO_2_. MRC-5 cells, a normal human fetal lung fibroblast cell line (ATCC), were maintained in Eagle’s minimal essential medium (ATCC) supplemented with 100 units/mL penicillin, 100 µg/mL streptomycin (Invitrogen, Carlsbad, CA, USA), and 10% fetal bovine serum (FBS, HyClone, Logan, UT, USA) at 37 °C in a humidified incubator containing 5% CO_2_. Cells were treated with 5 ng/mL TGF-β1 for 24 h in the presence or absence of 11,12-EET (Abcam, Cambridge, MA, USA), 1-trifluoromethoxyphenyl-3-(1-propionylpiperidin-4-yl) urea (TPPU, Sigma-Aldrich, St. Louis, MO, USA) or sulphaphenazole (Abcam). To isolate primary fibroblasts, IPF lung tissues were cut into 1 × 1 mm^2^ pieces and cultured at 37 °C in a humidified atmosphere containing 5% CO_2_ for 7‒10 days, the medium was changed every 3 days. Cells at passages 3 to 7 were used for all experiments. Detailed protocols for other experiments can be found in the online supplement.

### Statistical analysis

All values are represented as the mean ± standard error. Experiments were repeated at least three times, and the data were analyzed for statistical significance using GraphPad Prism 5 software (GraphPad Inc. La Jolla, CA, USA). For in vitro assays, significant differences were analyzed using one-way analysis of variance (ANOVA), followed by the Newman‒Keuls multiple comparison test when comparing >3 groups. Statistical analysis of eicosanoid levels in human lung tissues was performed using the Mann‒Whitney nonparametric test. Receiver operating characteristic (ROC) curve analysis was performed to confirm the optimal cutoff value of EETs for IPF diagnosis. All *p*-values were two-tailed, with statistical significance set at *p* < 0.05.

## Results

### EET expression is reduced in IPF lung tissues

**Fig. 1:**
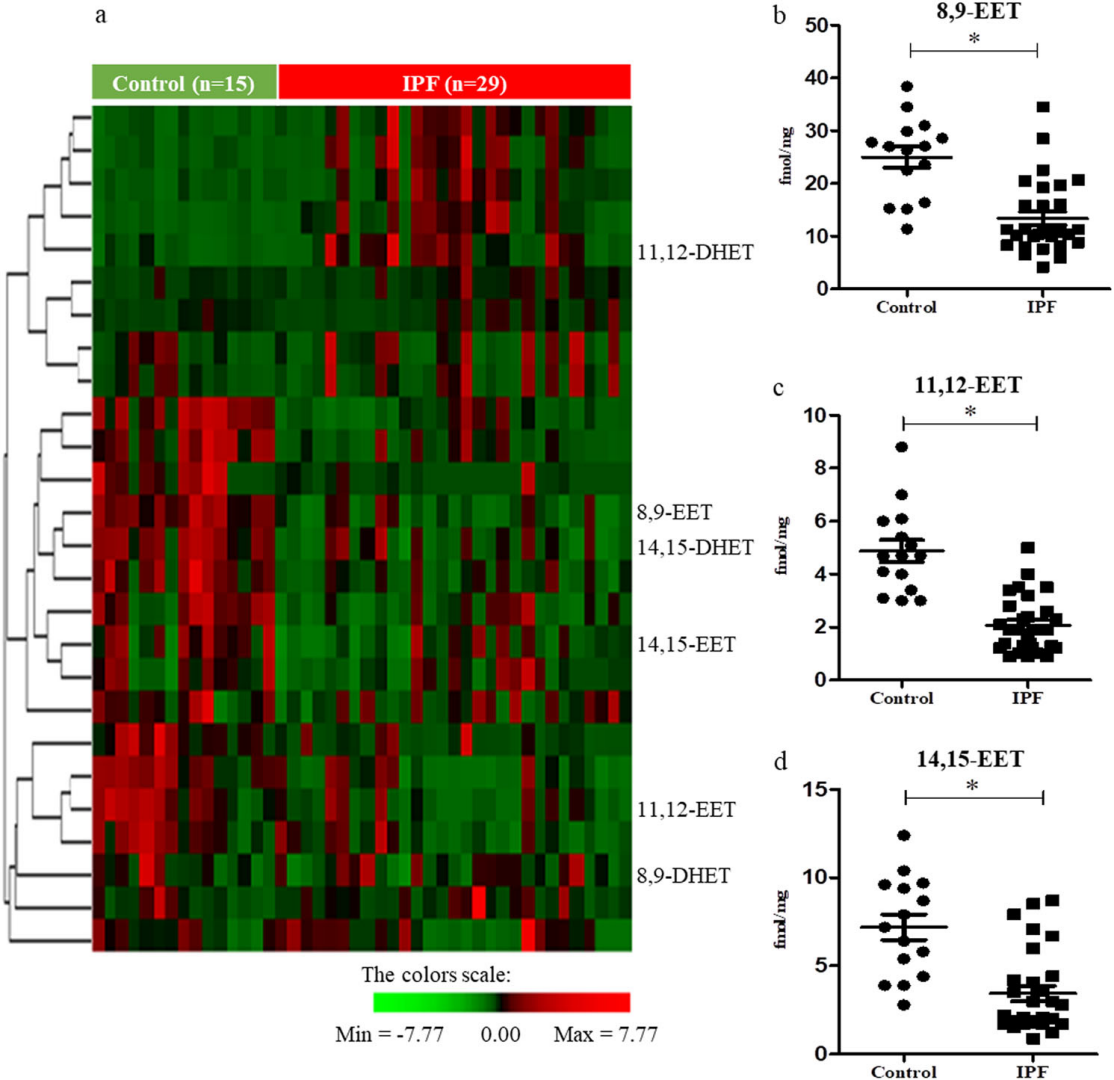
Epoxyeicosatrienoic acid levels are reduced in the lung tissues of patients with idiopathic pulmonary fibrosis. Quantification of arachidonic acid-related metabolites in human idiopathic pulmonary fibrosis (IPF; *n* = 29) or control lung tissues (*n* = 15) was performed using liquid chromatography with tandem mass spectrometry. **a** Heatmap visualization of arachidonic acid-related metabolites in human idiopathic pulmonary fibrosis or control lung tissues. Epoxyeicosatrienoic acids (EETs) and soluble epoxy hydrolase-related metabolites are labeled in the heatmap. **b**–**d** IPF lung tissues exhibited lower levels of EETs (8,9-EET [**b**], 11,12-EET [**c**], and 14,15-EET [**d**]) than control lung tissues. * indicates *p* < 0.0001 compared to control lung tissues.

The levels of eicosanoids in human IPF lung tissues (*n* = 29) and control tissues (*n* = 15) were measured using LC-MS/MS. The IPF group had lower lung function than the control group (Supplementary Table S1). Although the levels of several eicosanoids differed significantly between the IPF and control groups (Fig. 1a), EETs exhibited the most notable differences. The levels of EETs, including 8,9-EET (mean, 13.39 vs. 25.00 fmol/mg lung tissue, *p* < 0.001), 11,12-EET (mean, 2.07 vs. 4.87 fmol/mg lung tissue, *p* < 0.001), and 14,15-EET (mean, 3.44 vs. 7.19 fmol/mg lung tissue, *p* < 0.001), in IPF lung tissues were significantly lower than those in control lung tissues (Fig. 1b–d), and 4,5-EET was not detected. ROC curve analysis revealed that EETs were useful in discriminating IPF from control conditions; the optimal cutoff values of 8,9-EET, 11,12-EET, and 14,15-EET were 22.5 fmol/mg lung tissue (AUC 0.88, *p* < 0.001), 3.367 fmol/mg lung tissue (AUC 0.94, *p* < 0.001), and 4.285 fmol/mg lung tissue (AUC 0.87, *p* < 0.001), respectively, (Supplementary Fig. S1).

### Increased expression of sEH contributes to changes in EET levels in IPF lung tissues

**Fig. 2:**
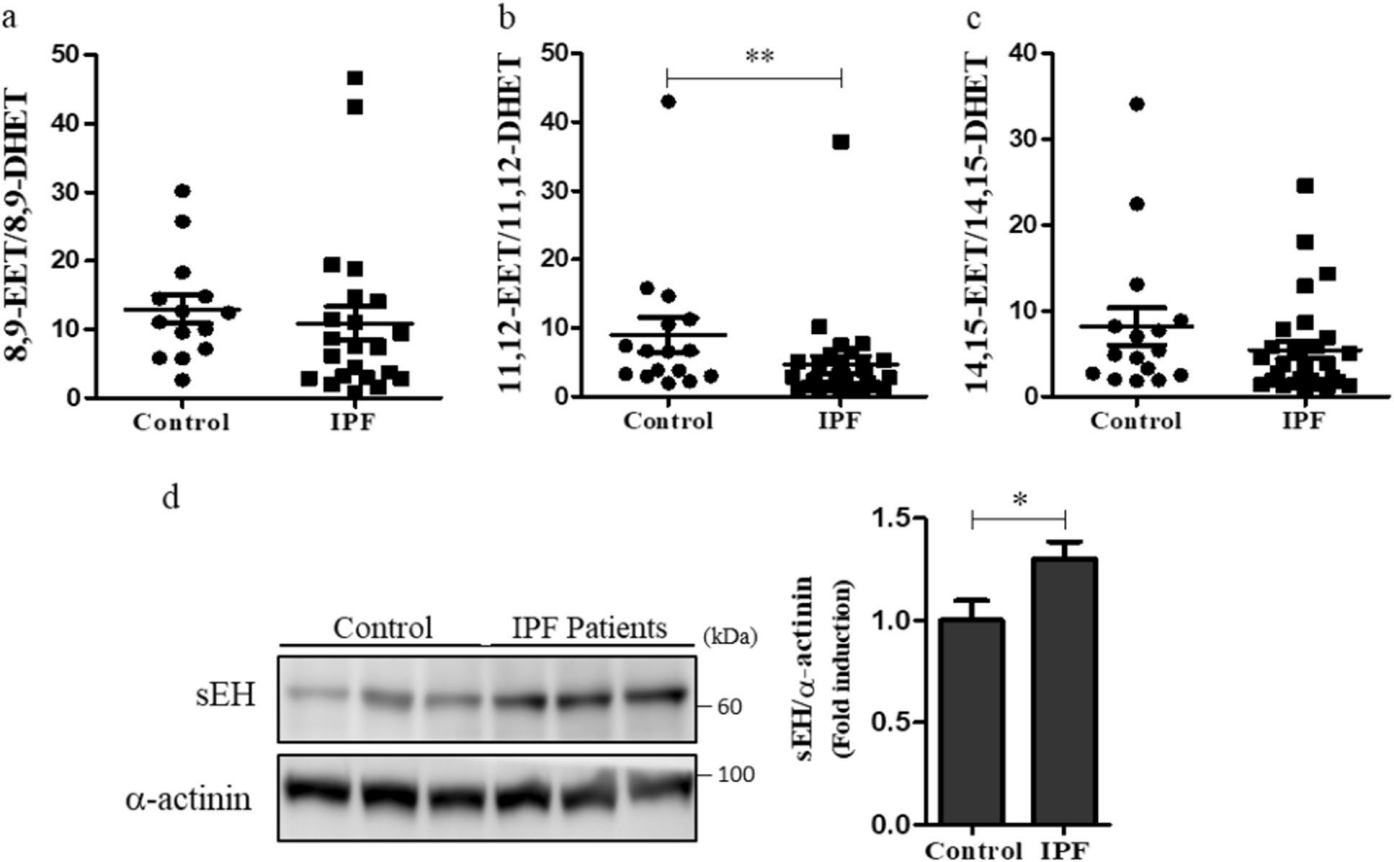
Soluble epoxide hydrolase levels are increased in the lung tissues of patients with idiopathic pulmonary fibrosis. **a**–**c** The ratio of 8,9-EET to 8,9-dihydroxyeicosatrienoic acid (DHET) (**a**), 11,12-EET to 11,12-DHET (**b**), and 14,15-EET to 14,15-DHET (**c**) in idiopathic pulmonary fibrosis (IPF) lung tissues compared with that in control lung tissues. ** indicates *p* < 0.01 compared to control lung tissues. **d** The levels of soluble epoxide hydrolase in IPF and control lung tissues were analyzed by western blotting. Densitometry was used to analyze fold changes in the levels of soluble epoxide hydrolase/α-actinin (*p* < 0.05). These data suggest that the reduced ratio of 11,12-EET to 11,12-DHET in IPF lung tissues might be due to the upregulated expression of soluble epoxide hydrolase.

As EETs are rapidly metabolized into their corresponding diol forms by sEH, we measured the levels of DHETs. While the levels of 8,9-DHET and 11,12-DHET did not show significant differences between the two groups, the levels of 14,15-DHET in IPF lung tissues were significantly lower than those in the control (Supplementary Fig. S2). We also determined the activity of sEH by measuring the ratio of EETs to DHETs. The ratio of 11,12-EET/11,12-DHET in IPF lung tissues was significantly lower than that in the control (mean, 4.63 vs. 9.00 *p* < 0.01, Fig. 2b), while the ratios other EETs did not exhibit any significant differences between the two groups (Fig. 2a, c). We next measured the expression of sEH in lung tissues. The levels of sEH were significantly higher in IPF lung tissues than in control tissues (Fig. 2d). Our data suggest that the reduced levels of 11,12-EET in IPF lung tissues might be due to the upregulation of sEH.

### 11,12-EET and sEH inhibition suppresses fibroblast activation

TGF-β1 induces epithelial cell injury and stimulates fibroblast activation^24^. In MRC-5 cells, which are human lung fibroblasts, TGF-β1 increased the expression of α-SMA and collagen type 1, and 11,12-EET significantly reduced the TGF-β1-induced expression of these proteins (Supplementary Fig. S3a, b). We next examined the effects of the sEH inhibitor TPPU, which increased the levels of EETs. TPPU significantly reduced the TGF-β1-induced expression of α-SMA and collagen type 1 in MRC-5 cells (Supplementary Fig. S3c). Under identical conditions, 11,12-EET and TPPU did not significantly affect the cytotoxicity (Supplementary Fig. S4a, b) or viability (Supplementary Fig. S4e, f) of MRC-5 cells.

To ascertain whether sEH activity was essential for fibroblast activation, we investigated the effects of sEH knockdown using specific siRNA. MRC-5 cells were transfected with control siRNA or sEH-specific siRNA for 48 h and then treated with TGF-β1 for 24 h. TGF-β1 upregulated the expression of sEH, and sEH-specific siRNA effectively downregulated the basal expression or TGF-β1-induced expression of sEH (Supplementary Fig. S3d). sEH knockdown significantly inhibited the TGF-β1-induced expression of α-SMA and collagen type 1 (Supplementary Fig. S3d).

**Fig. 3:**
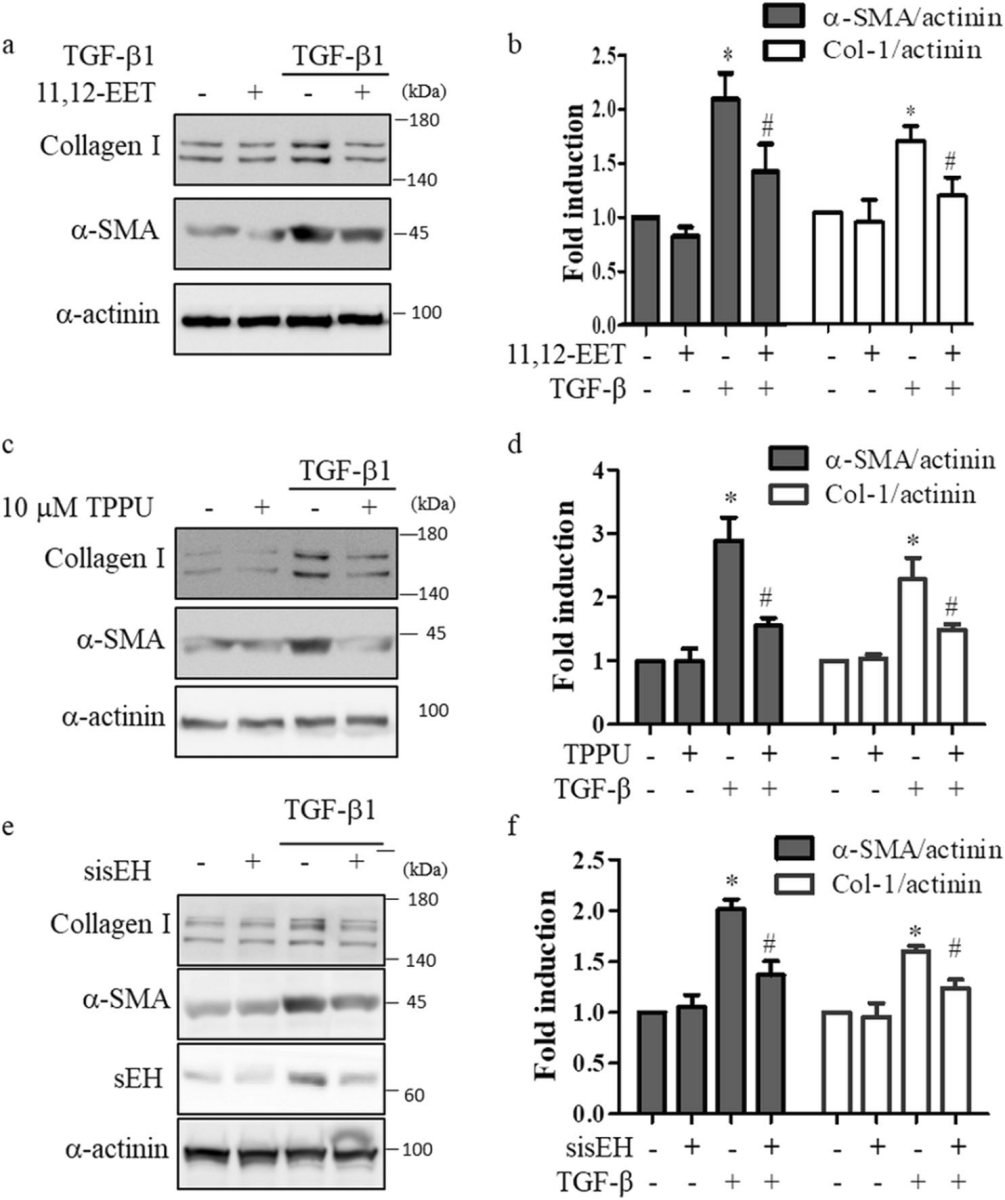
TGF-β1-induced activation of primary human lung fibroblasts is suppressed by 11,12-EET and the inhibition of sEH. **a** Primary human lung fibroblasts were treated with the indicated concentration of 11,12-EET and then stimulated with 5 ng/mL TGF-β1 for 24 h. Total cell extracts were prepared and subjected to western blotting using antibodies against collagen type 1, α-smooth muscle actin, and α-actinin. Representative immunoblots are shown. **b** Densitometry was used to analyze fold changes in the levels of α-smooth muscle actin (α-SMA) and collagen type 1 (Col-1). **c** Primary human lung fibroblasts were treated with the indicated amount of TPPU and then stimulated with 5 ng/mL TGF-β1 for 24 h. Total cell extracts were prepared and subjected to western blotting. **d** Densitometry was used to analyze fold changes in the levels of α-smooth muscle actin (α-SMA) and collagen type 1 (Col-1). **e** Primary human lung fibroblasts were transfected with control or soluble epoxide hydrolase siRNA for 48 h. Cells were treated with 5 ng/mL TGF-β1 for 24 h, and total cell extracts were prepared and subjected to western blotting. **f** Densitometry was used to analyze fold changes in the levels of α-smooth muscle actin (α-SMA) and collagen type 1 (Col-1). * indicates *p* < 0.05 compared to the control, and # indicates *p* < 0.05 compared to TGF-β1 treatment alone.

We confirmed our results in primary fibroblasts from patients with IPF. Consistent with the results in MRC-5 cells, 11,12-EET significantly reduced the TGF-β1-induced expression of α-SMA and collagen type 1 in primary fibroblasts (Fig. 3a, b). In addition, the inhibition of sEH by TPPU (Fig. 3c, d) and sEH-specific siRNA (Fig. 3e, f) reduced the effects of TGF-β1 on primary fibroblasts. These results suggest that 11,12-EET and sEH inhibition may reduce fibroblast activation.

### 11,12-EET and sEH inhibition suppresses TGF-β1-induced Smad signaling in fibroblasts

**Fig. 4:**
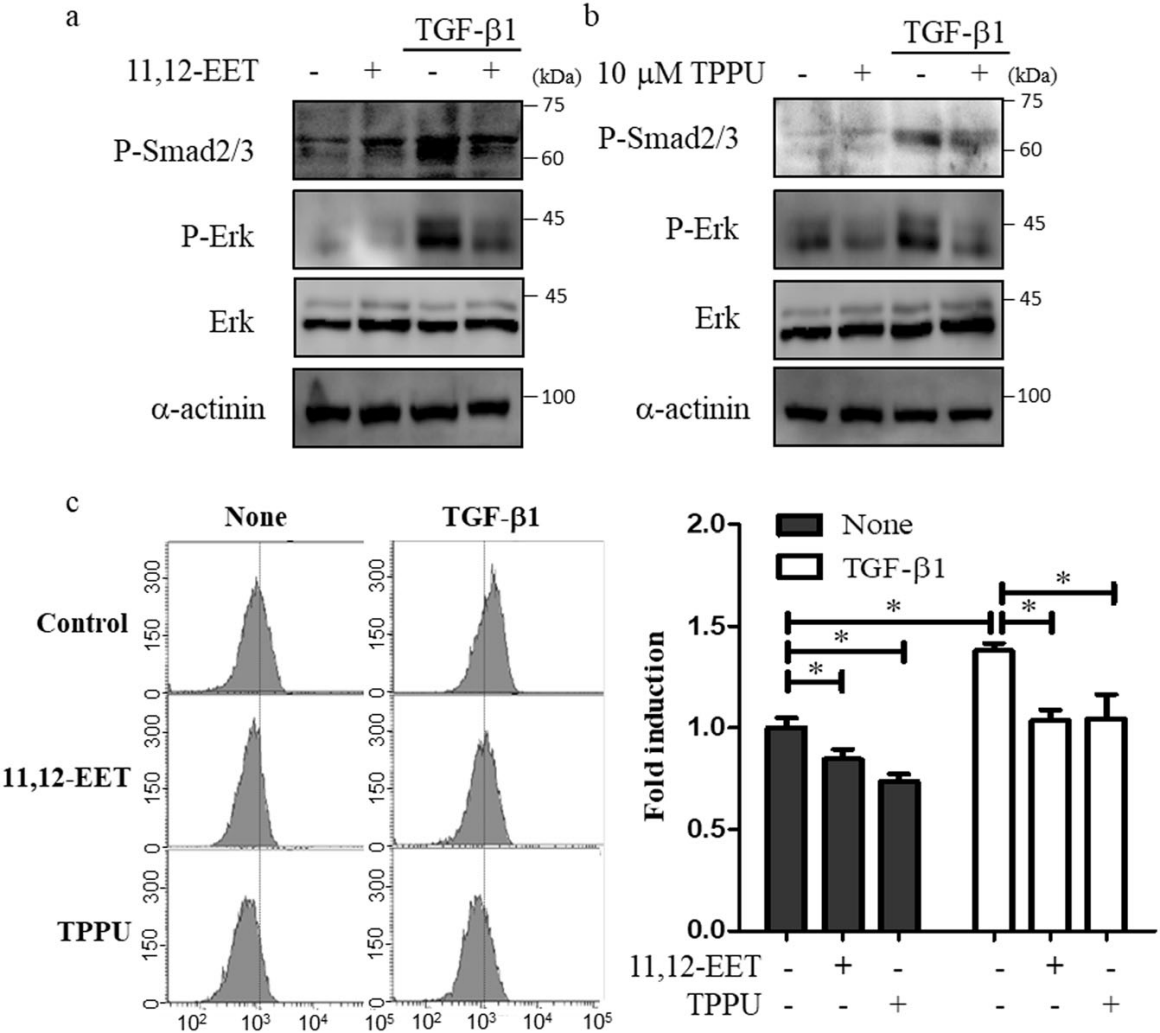
Inhibition of TGF-β1-induced phosphorylation of Smad2/3 and extracellular-signal-regulated kinase and reduced reactive oxygen species (ROS) levels in primary human lung fibroblasts in response to treatment with 11,12-EET and TPPU. **a** Primary human lung fibroblasts were exposed to 5 ng/mL TGF-β1 for 1 h with/without 1 µM 11,12-EET. **b** Primary human lung fibroblasts were pretreated with 10 µM TPPU for 23 h and then stimulated with 5 ng/mL TGF-β1 for 1 h. Total cell extracts were prepared and subjected to western blotting using antibodies against p-Smad2/3, p-Erk, Erk, and α-actinin. **c** Primary human lung fibroblasts were pretreated with 1 µM 11,12-EET for 1 h or 10 µM TPPU for 23 h and then stimulated with 5 ng/mL TGF-β1 for 1 h. ROS levels were measured using 2’,7’-dichlorodihydrofluorescein diacetate dye. The data are expressed as the mean values of triplicate experiments. * and ** indicate *p* < 0.05 and *p* < 0.01, respectively.

TGF-β1 stimulates the Smad signaling pathway via the phosphorylation of Smad2/3^25^ and activates the extracellular-signal-regulated kinase (ERK) pathway^26^. In this study, TGF-β1 increased the phosphorylation of Smad2/3 and ERK in primary fibroblasts from patients with IPF, and treatment with 11,12-EET and TPPU suppressed these effects (Fig. 4a, b). Moreover, as oxidative stress may contribute to pulmonary fibrosis^27^, we measured the levels of reactive oxygen species (ROS) and found that 11,12-EET and TPPU reduced TGFβ1-induced ROS levels in primary fibroblasts (Fig. 4c).

### Inhibition of CYP activity increases fibroblast activation

**Fig. 5:**
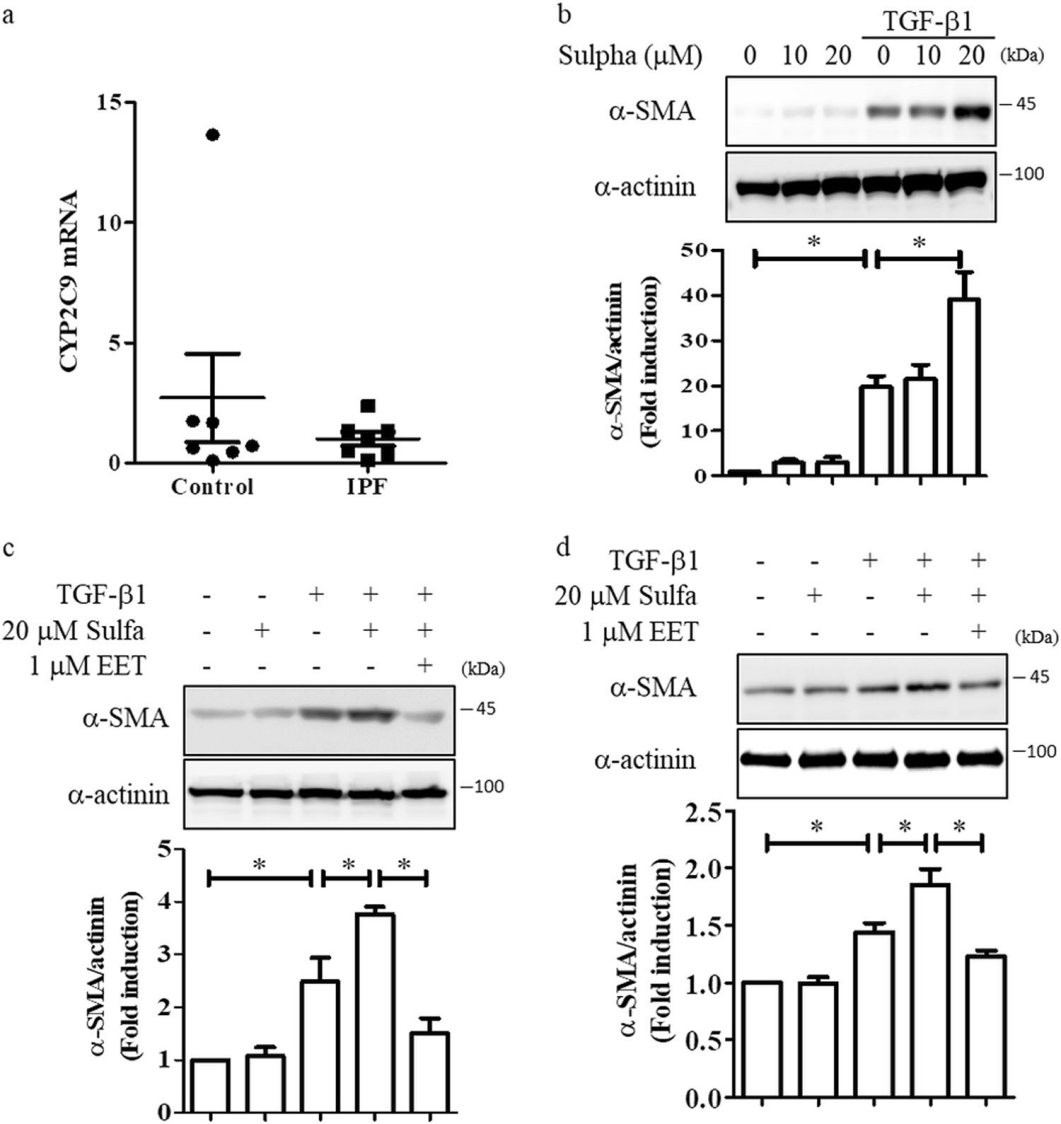
Sulphaphenazole enhances the TGF-β1-induced increase in α-smooth muscle actin levels in human lung fibroblasts. **a** The mRNA levels of *CYP2C9* in human idiopathic pulmonary fibrosis (IPF; *n* = 7) or control lung tissues (*n* = 7) were measured by real-time polymerase chain reaction. **b** MRC-5 cells were treated with the indicated amount of sulphaphenazole (Sulpha) and then stimulated with 5 ng/mL TGF-β1 for 24 h. Densitometry was used to analyze fold changes in the levels of α-smooth muscle actin (α-SMA). **c** MRC-5 cells were stimulated with 5 ng/mL TGF-β1 for 24 h with/without 20 µM sulphaphenazole and 1 µM 11,12-EET. Densitometry was used to analyze fold changes in the levels of α-smooth muscle actin (α-SMA). **d** Primary human lung fibroblasts were stimulated with 5 ng/mL TGF-β1 for 24 h with/without 20 µM sulphaphenazole and 1 µM 11,12-EET. Total cell extracts were prepared and subjected to western blotting using antibodies against α-smooth muscle actin and α-actinin. Densitometry was used to analyze fold changes in the levels of α-smooth muscle actin (α-SMA). * indicate *p* < 0.05.

The most abundant cytochrome P450s (CYPs) in human lung tissue are CYP2C9, CYP1B1, CYP2B6, and CYP2E1^28^. CYP2C9 is a good target for studying EET metabolism in the lung, as CYP2C and CYP2J metabolize arachidonic acid into EETs. Therefore, we investigated whether CYP2C9 activity was involved in fibroblast activation. The mRNA levels of CYP2C9 did not show significant differences between human IPF and control lung tissues (Fig. 5a). Sulphaphenazole, a CYP2C9 inhibitor, dose-dependently enhanced TGF-β1-induced expression of α-SMA in MRC-5 cells (Fig. 5b). To examine whether the effects of CYP2C9 inhibition were dependent on the levels of 11,12-EET, we investigated the effects of 11,12-EET on the TGF-β1- and sulphaphenazole-induced upregulation of α-SMA. 11,12-EET reduced the TGF-β1- and sulphaphenazole-induced upregulation of α-SMA in MRC-5 cells (Fig. 5c) and primary fibroblasts from patients with IPF (Fig. 5d).

### 11,12-EET reduces the expression of fibronectin in Beas-2B cells

**Fig. 6:**
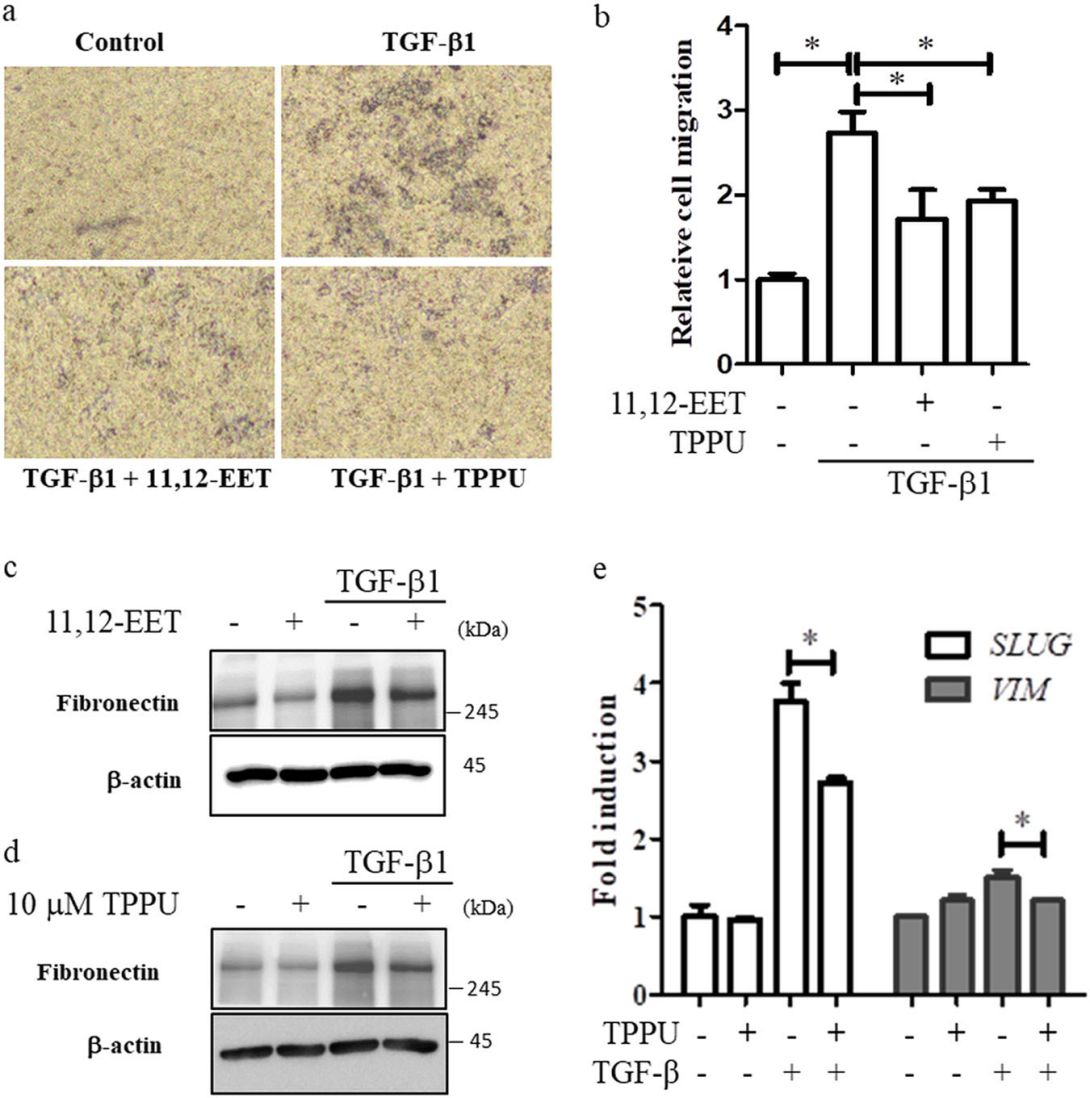
Reduced expression of fibronectin in epithelial cells in response to 11,12-EET and TPPU. **a**, **b** The migrated cells on the lower surface of the upper part of the transwell membrane were stained with 0.2% crystal violet and observed using a phase-contrast microscope (**a**). The stain was eluted, and the absorbance was measured at 540 nm (**b**). **c**, **d** Beas-2B cells were treated with 5 ng/mL TGF-β1 for 24 h with/without 1 μM 11,12-EET (**c**) or 10 μM TPPU (**d**). Total cell extracts were prepared and subjected to western blotting using antibodies against fibronectin and β-actin. **e** Beas-2B cells were treated with 5 ng/mL TGF-β1 for 24 h with/without 10 μM TPPU. The mRNA levels of *SLUG* and *VIM* were determined by real-time polymerase chain reaction. * indicates *p* < 0.01.

To evaluate the effect of 11,12-EET on epithelial-mesenchymal transition (EMT) in the context lung fibrosis^29^, a migration assay was performed in Beas-2B cells. Beas-2B cells were treated with 11,12-EET or TPPU for 48 h, which resulted in significant reductions in TGF-β1-induced cell migration (Fig. 6a, b). To confirm the antifibrotic effect, we analyzed the expression of the epithelial cell marker E-cadherin and the mesenchymal cell marker fibronectin in TGF-β1-treated Beas-2B cells. The increased levels of fibronectin in TGF-β1-treated Beas-2B cells were reduced in response to 11,12-EET and TPPU treatment (Fig. 6c, d), whereas sulphaphenazole did not exert any effect (Supplementary Fig. S5c). However, 11,12-EET, TPPU, and sulphaphenazole did not affect the reductions in the levels of E-cadherin in TGF-β1-treated Beas-2B cells (Supplementary Fig. S5). Under identical conditions, 11,12-EET and TPPU did not affect the cytotoxicity (Supplementary Fig. S4c, d) or viability (Supplementary Fig. S4g, h) of Beas-2B cells. We next analyzed the mRNA expression of *SLUG* and *VIM*, which are TGF-β1-induced EMT-related genes^30,31^. TPPU significantly inhibited TGF-β1-induced mRNA levels of *SLUG* and *VIM* (Fig. 6e). Our results suggest that 11,12-EET and TPPU may exert antifibrotic effects on epithelial cells by reducing the expression of fibronectin.

### TPPU attenuates pulmonary fibrosis in a bleomycin-induced mouse model

**Fig. 7:**
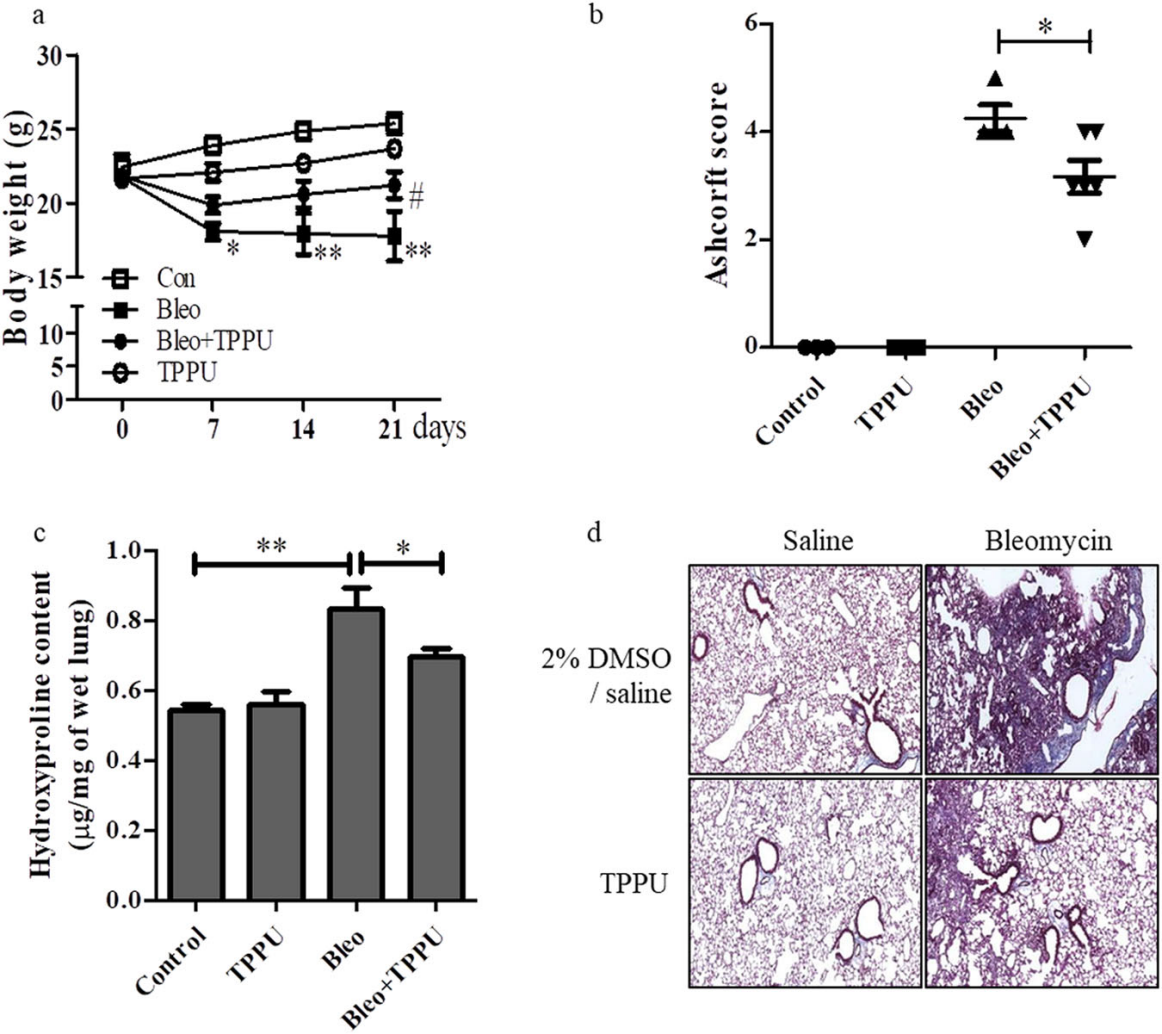
The effects of TPPU on the bleomycin-induced pulmonary fibrosis mouse model. **a** TPPU (0.25 mg/kg) was administered to mice 5 days/week for 3 weeks after bleomycin administration (3 U/kg). Body weights were analyzed in the four groups of mice: control (*n* = 3), bleomycin treatment (*n* = 4), TPPU treatment (*n* = 3), and bleomycin + TPPU treatment (*n* = 6). * and ** indicate *p* < 0.05 and *p* < 0.01, respectively, compared to the control group. # indicates *p* < 0.05 compared to the bleomycin treatment group. **b** Lung fibrosis scores were quantified by a modified Ashcroft scale (grade 0~8). **c** Collagen content was estimated by hydroxyproline assays. * and ** indicate *p* < 0.05 and *p* < 0.01, respectively (one-way ANOVA test with the Newman‒Keuls multiple comparison test). **d** Representative histological lung sections from each group stained with Masson’s trichrome.

To determine whether the in vitro effects of TPPU could be recapitulated in vivo, we evaluated the antifibrotic effects of TPPU on a bleomycin-induced mouse model. After intratracheal injection of bleomycin (3 U/kg), 0.25 mg/kg TPPU was immediately administered to the mice 5 days per week for 3 weeks by intraperitoneal injection, and the mice were euthanized after 21 days. The bleomycin-induced group showed severe weight loss after bleomycin administration compared to that of the control group; however, TPPU treatment significantly attenuated weight loss in the bleomycin-induced group (Fig. 7a). Histopathological examination of lung sections (Supplementary Fig. S6) and the fibrotic lesion scores (Fig. 7b) showed that TPPU prevented the development of bleomycin-induced pulmonary fibrosis. Mice treated with bleomycin showed significantly higher levels of collagen, as measured by hydroxyproline assays (Fig. 7c) and Mason’s trichrome staining (Fig. 7d), in lung tissues than control mice, while TPPU significantly attenuated bleomycin-induced increases in collagen levels. In summary, these data suggest that sEH inhibition by TPPU is a promising therapeutic strategy for pulmonary fibrosis.

## Discussion

In the present study, we demonstrated changes in the levels of eicosanoids, including EETs, in IPF lung tissues. Among the EETs, the levels of 11,12-EET were the most significantly different between the IPF and control groups. 11,12-EET reduced the TGF-β1-induced activation of fibroblasts and epithelial cell EMT. TPPU also showed antifibrotic effects both in vitro and in vivo. These data suggest that 11,12-EET and the regulation of its metabolic pathway prevent pulmonary fibrosis.

The effect of EETs or sEH inhibitors on pulmonary fibrosis is associated with some signaling pathways, such as the Smad, p38 MAPK, and EMT-related signaling pathways^29,32^. In our study, the levels of EETs in IPF lung tissues were lower than those in control lung tissues. Therefore, we hypothesized that the dysregulation of EET-related signaling was associated with the development of pulmonary fibrosis. We found that 11,12-EET or sEH inhibitors downregulated TGF-β1-induced Smad2/3 and ERK pathways in TGF-β1-treated fibroblasts and downregulated EMT in TGF-β1-treated Beas-2B cells. Thus, our results suggest that the EET-related metabolic pathway can serve as a therapeutic target for IPF treatment.

Recent studies suggested that IPF develops as a result of an aberrant wound healing response after recurrent alveolar injury^2^ and that dysregulation of lipid metabolism is involved in the pathogenesis of IPF^6,7,31^. EETs serve as important antifibrotic lipid mediators in fibrotic diseases such as pulmonary and renal fibrosis^17,18,33^. In our study, EET levels were reduced in the lung tissues of patients with IPF compared to control lung tissues. This reduction may be due to the dysregulated activity of two key enzymes, sEH and CYP2C9, in the biosynthetic pathway of EETs. sEH, a key enzyme that is responsible for metabolizing EETs into DHETs, is widely distributed in mammalian tissues, including the lungs^34^. The inhibition of sEH activity by a pharmacological inhibitor or genetic disruption can induce the accumulation of EETs and exert protective effects against lung, liver, and renal fibrosis^14–17^. In our study, increased expression of sEH was observed in IPF lung tissues, and TPPU-mediated inhibition of sEH activity effectively reduced lung fibrosis in in vitro and in vivo in lung fibrosis models. Overall, our data suggest that the regulation of EET-related enzymes may be an important strategy in the treatment of IPF.

Additionally, the hydration of EETs by epoxide hydrolase is regioselective^35^. While 5,6-EET is a poor substrate of sEH, 8,9-EET, 11,12-EET and 14,15-EET are effectively utilized by sEH^36^. Moreover, among 8,9-EET, 11,12-EET and 14,15-EET, the hydration rate of 11,12-EET by lung cytosolic epoxide hydrolase is the lowest^35^. However, in the present study, we only observed a reduction in the 11,12-EET/11,12-DHET ratio in IPF lung tissues, and the 8,9-EET/8,9-DHET and 14,15-EET/14,15-DHET ratios were not significantly different between the IPF and control lung tissues. These results might be associated with the increases in 8,9-DHET and 14,15-DHET levels, while higher hydration rates for a long time might cause 8,9-EET and 14,15-EET to act as poor substrates of sEH to maintain homeostasis or might be associated with the excretion ratio of diol products.

CYP2C9 is one of the CYPs expressed in human lung tissues^28^ that metabolizes arachidonic acid into EETs, thereby eliciting various physiological responses, including antifibrotic effects^17,37^. The production of 11,12-EET by CYP2C9 in endothelial cells induces the expression of COX-2^38^, and the COX-2-PGE2 axis exerts an antifibrotic effect by promoting the survival of epithelial cells and the death of fibroblasts/myofibroblasts in IPF^3^, suggesting that CYP2C9 may be involved in the pathogenesis of IPF. However, the role of CYP2C9 in lung fibrosis is incompletely understood. We therefore tested the hypothesis that sulphaphenazole-mediated inhibition of CYP2C9 could reduce the production of 11,12-EET and exacerbate lung fibrosis. We found that sulphaphenazole increased the TGF-β1-induced upregulation of α-SMA in human lung fibroblasts, which was reduced by treatment with 11,12-EET. These data suggest that 11,12-EET production might increase due to the activity of CYP2C9, thereby reducing the activation of human lung fibroblasts via the regulation of the COX-2-PGE2 axis.

In conclusion, our data suggest that 11,12-EET and sEH inhibition may prevent pulmonary fibrosis by inhibiting the activation of fibroblasts and epithelial cell EMT. Therefore, the regulation of EETs might serve as a therapeutic strategy for IPF treatment.

## Supplementary information

supplment figure and tables
