# Supplementary material for: The arachidonic acid metabolite 11,12-epoxyeicosatrienoic acid alleviates pulmonary fibrosis
Source: Exp Mol Med. 2021 May 14;53(5):864–74. doi: 10.1038/s12276-021-00618-7 (PMC8178404; doi:10.1038/s12276-021-00618-7)
Supplement: Supplementary file 1 — supplment figure and tables [file 12276_2021_618_MOESM1_ESM.docx]

**Arachidonic acid metabolite, 11,12-epoxyeicosatrienoic acid, alleviates pulmonary fibrosis**

Hak Su Kim^1,2^, Su-Jin Moon^1^, Sang Eun Lee^1^, Gi Won Hwang^1^, Hyun Ju Yoo^3^, Jin Woo Song^1^

^1^Department of Pulmonary and Critical Care Medicine, Asan Medical Institute of Convergence Science and Technology, Asan Medical Center, University of Ulsan College of Medicine, Seoul, Republic of Korea

^2^Veterans Medical Research Institute, Veterans Health Service Medical Center, Seoul, Republic of Korea

^3^Department of Convergence Medicine, Asan Medical Institute of Convergence Science and Technology, Asan Medical Center, University of Ulsan College of Medicine, Seoul, Republic of Korea

**Online Supplementary Material**

**Materials and Methods**

**Analysis of eicosanoids by LC-MS/MS**

Eicosanoids were extracted from human lung tissues (~20 mg) using solid phase extraction (SPE). Sorbent per Oasis HLB SPE cartridge (60 mg, Waters, Milford, MA, USA) was washed and preconditioned with ethylacetate, methanol, and 0.1% acetic acid:5% methanol in H_2_O, sequentially. Ten microliters of EDTA (0.2 mg/mL) and BHT in MeOH: H_2_O (50:50) were loaded onto the sorbent bed of an SPE column. PGD_2_-d_4_, PGE_2_-d_4_, 9(10)-EpOME-d_4_, and CUDA were also added as internal standards to the samples. Sample solutions were loaded onto the SPE column. The column was washed with 2 column volumes of 0.1% acetic acid:5% methanol in H_2_O, then the column was dried using vacuum. Finally, eicosanoids were eluted with 0.5 mL of methanol followed by 1.5 mL of ethylacetate. Sample solutions were dried using vacuum centrifuge, and the dried matter was reconstituted with 40 μL of 50% ACN prior to a liquid chromatography with tandem mass spectrometry (LC-MS/MS). A LC-MS/MS system equipped with 1290 HPLC (Agilent), Qtrap 5500 (ABSciex) and reverse phase column (Pursuit 5 200 × 2.0 mm) was used. The separation gradient for PGD_2_ and PGE_2_ used mobile phase A (0.1% acetic acid in H_2_O) and mobile phase B (0.1% acetic acid in ACN/MeOH (84/16, v/v) and proceeded at 300 µL/min and 25°C. The following separation gradient was used: 35 to 45% B for 1 min, 45 to 55% B for 2 min, 55 to 66% B for 5.5 min, 66 to 72% B for 4 min, 72 to 82% B for 2.5 min, 82 to 95% B for 1 min, hold at 95% B for 1.5 min, 95 to 35% B for 0.1 min, and then hold at 35% B for 3.15 min. The multiple reaction monitoring (MRM) mode was used in the negative ion mode, and the ion chromatogram corresponding to the specific transition of each analyte was used for quantification. The calibration range for each analyte was 0.1−1000 nM (r^2^ ≥ 0.99). Data analysis was performed using Analyst 1.5.2 software (SCIEX, Framingham, MA, USA).

**Western blotting**

Proteins were extracted from lung homogenates or cell lysates using radioimmune precipitation assay buffer (50 mM Tris-HCl, pH 7.5; 1% Triton X-100; 150 mM NaCl; 1% sodium deoxycholate; 2 mM EDTA; and 0.1% sodium dodecyl sulphate [SDS]), and separated by SDS-polyacrylamide gel electrophoresis and transferred to polyvinylidene difluoride or nitrocellulose membranes (Pall Corporation, Port Washington, NY, USA) in transfer buffer. Primary antibodies against sEH (Santa Cruz Biotechnology, Dallas, TX, USA), α-smooth muscle actin (SMA, Abcam, Cambridge, UK), collagen type 1 (Abcam), α-actinin (Santa Cruz Biotechnology), E-cadherin (5H9, Santa Cruz Biotechnology), p-Smad2/3 (Ser 423/425, Santa Cruz), and P-Erk (Cell Signalling Technology, Boston, MA, USA) were used. Goat anti-rabbit, goat anti-mouse, or rabbit anti-goat horseradish peroxidase-conjugated antibodies (Sigma-Aldrich, St Louis, MO, USA) were used as secondary antibodies. Immunoreactive bands were visualised using enhanced chemiluminescence detection system (Pierce) and analysed using Quantity One image analysis software (Bio-Rad Laboratories, Hercules, CA, USA).

**Knockdown of sEH by sEH–siRNA**

sEH-siRNA (h) (sc-44090) and control siRNA (sc-37007) were purchased from Santa Cruz Biotechnology (Santa Cruz, CA, USA). MRC-5 cells and primary human fibroblasts, from patients with IPF, were transfected with sEH-siRNA or control siRNA using X-tremeGENE^TM^ siRNA Transfection Reagent (Roche, Indianapolis, IN, USA) according to the manufacturer’s instructions.

**Measurement of reactive oxygen species**

The cells were exposed to 1 μM of 11,12-EET or 10 μM of TPPU for 1 h and incubated with 10 μM 2',7'–dichlorofluorescin diacetate (DCF-DA) for 30 min. The adherent cells were trypsinised and collected in a 15 mL tube. After two washes with phosphate buffered saline (PBS, pH 7.4), the intensity of DCF-DA fluorescence was determined on FACSCanto™II (BD Bioscience, San Jose, CA, USA) using an excitation wavelength of 480 nm and an emission wavelength of 530 nm.

**Reverse transcription PCR and quantitative real-time PCR**

Total RNA from harvested cells was isolated using TRIzol reagent (Invitrogen, Carlsbad, CA, USA) and reverse transcribed into cDNA. The amplification was performed on an ABI StepOnePlus^TM^ Real-Time polymerase chain reaction (PCR) thermal cycler using Power SYBR Green PCR Master Mix according to the manufacturer’s protocol (Applied Biosystems, CA, USA). Target mRNA levels were normalised against β-actin, and the relative mRNA expression levels were calculated.

**Cytotoxicity and viability assay**

Cytotoxicity was assessed using a lactate dehydrogenase (LDH) assay. Cells were grown in a 96-well culture plate in density of 3000 cells per well and were exposed to TGF-β1 for 24 hour in the presence of 11,12-EET or TPPU. Cells treated with lysis buffer supplied from Quanti-LDH cytotoxicity assay kit (BIOMAX, Seoul, South Korea) were acted as maximum LDH activity controls. 10 μL of cultured medium were mixed with 100 μL of reaction mixture at room temperature for 30 min keeping in a dark place. Subsequently, 10 μL of stop solution was added. The absorbance was measured at 450 nm and 650 nm wavelength respectively. Cell viability was assessed using an MTT assay. Cells were seeded in 96-well plates and were exposed to TGF-β1 for 24 hour in the presence of 11,12-EET or TPPU. The cells were treated with 5 g/ml MTT solution and incubated for 2 hours. The supernatant was gently removed and cells were dissolved in dimethyl sulfoxide. Absorbance was measured at 570 nm.

**Cell migration assay**

The migration assay was performed using a 24-well transwell unit with polycarbonate filters of 0.8 μm pore size (Corning Costar, Cambridge, MA). The lower part of the transwell was filled with DMEM plus 10% FBS as a chemoattractant. Beas-2B cells were suspended in serum-free DMEM with TGF-β1, 11,12-EET or TPPU, added to the upper part of transwell and incubated for 48 h. The cells that attached to the upper surface of the polycarbonate filter were completely removed by wiping with a cotton swab, and the filters were stained with a 0.2% crystal violet/20% methanol solution (Sigma-Aldrich, St. Louis, MO, USA). Stained filters observed with a phase-contrast microscope and then the stain was eluted, and absorbance was measured at 540 nm.

**Supplementary Table**

Supplementary Table S1. Comparison of baseline characteristics between patients with IPF and controls

|  | IPF | Control | p-value |
| --- | --- | --- | --- |
| Number of patients | 29 | 15 |  |
| Age, years | 64.3 ± 5.9 | 68 ± 7.2 | 0.099 |
| Sex (male/female) | 17/12 | 15/0 | 0.003 |
| FVC % predicted | 68.6 ± 14.3 | 95.1 ± 15.3 | <0.001 |
| DL_CO_ % predicted | 55.4 ± 16.0 | 97.4 ± 33.0 | <0.001 |
| TLC % predicted | 68.4 ± 12.4 | 98.1 ± 15.2 | <0.001 |

Data are presented as mean ± standard deviation or number unless otherwise indicated. IPF, idiopathic pulmonary fibrosis; FVC, forced vital capacity; DL_CO_, diffusing capacity of the lung for carbon monoxide; TLC, total lung capacity

**Supplemental Figure Legends**

Supplementary Fig. S1. The receiver operating characteristics curve for EETs discriminating IPF and control groups.


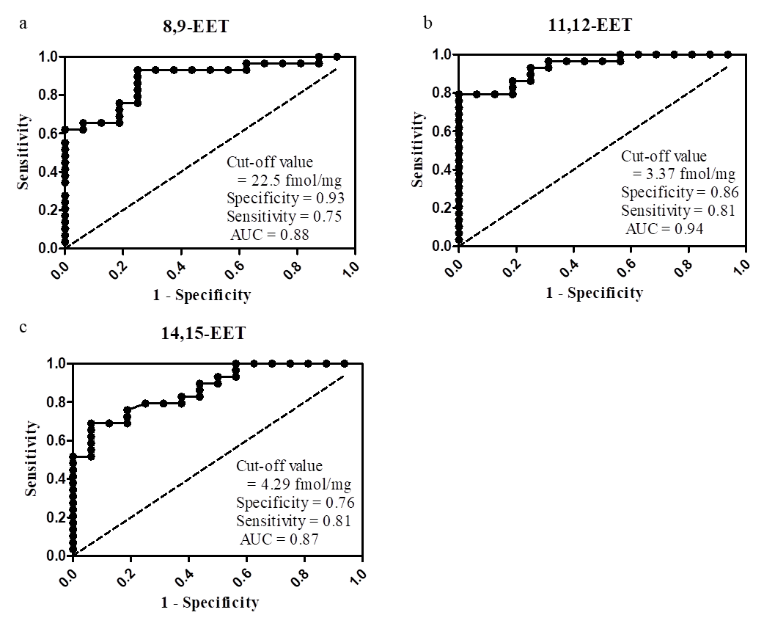


Quantification of arachidonic acid-related metabolites in idiopathic pulmonary fibrosis (IPF; n = 29) or control human lung tissues (n =15) was performed using liquid chromatography along with tandem mass spectrometry. a-c. The receiver operating characteristic curves for 8,9-EET (a), 11,12-EET (b), or 14,15-EET (c), for enabling discrimination between IPF and control groups.

Supplementary Fig. S2. The levels of DHETs in the lung tissues of patients with idiopathic pulmonary fibrosis.


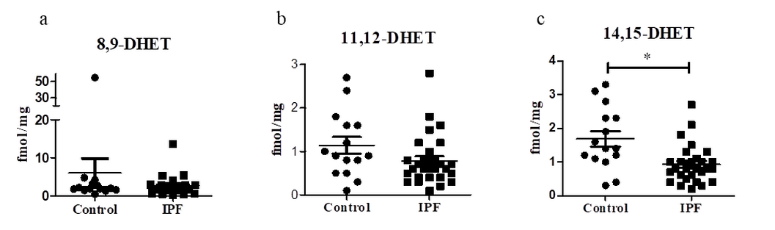


Quantification of DHETs in idiopathic pulmonary fibrosis (IPF; n = 29) or control human lung tissues (n =15) was performed using liquid chromatography along with tandem mass spectrometry. (a-c) The levels of 8,9-DHET (a), 11,12-DHET (b), 14,15-DHET (c) in IPF lung tissues were compared with in control lung tissues. * indicates p < 0.05 compared to control lung tissues.

Supplementary Fig. S3. TGF-β1-induced activation of human lung fibroblasts is inhibited by 11,12-EET and inhibition of sEH.


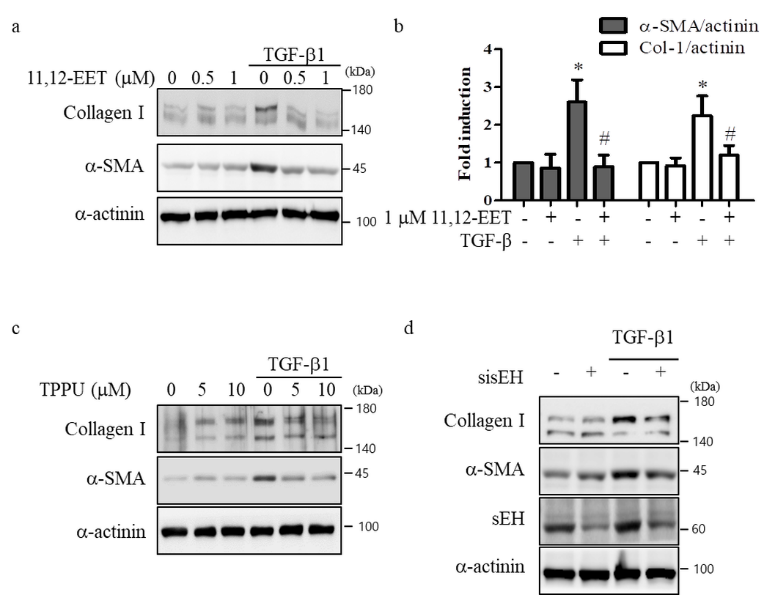


a. MRC-5 cells were treated with the indicated concentration of 11,12-EET and then stimulated with 5 ng/mL of TGF-β1 for 24 h. Total cell extracts were prepared and subjected to western blotting using antibodies against collagen type 1, α-smooth muscle actin, and α-actinin. Representative immunoblots are shown. b. Densitometry was used to analyse fold changes in the levels of α-smooth muscle actin and collagen type 1 (Col-1). * indicates p < 0.05 compared to control, and # indicates p < 0.05 compared to TGF-β1 treatment alone. c. MRC-5 cells were treated with the indicated amount of TPPU and then stimulated with 5 ng/mL of TGF-β1 for 24 h. Total cell extracts were prepared and subjected to western blotting. d. MRC-5 cells were transfected with control or soluble epoxide hydrolase siRNA for 48 h. Total cell extracts were prepared after treatment with 5 ng/mL of TGF-β1 for 24 h and subjected to western blotting.

Supplementary Fig. S4. Effect of 11,12-EET and TPPU on the cytotoxicity and viability of human lung fibroblasts and epithelial cells.


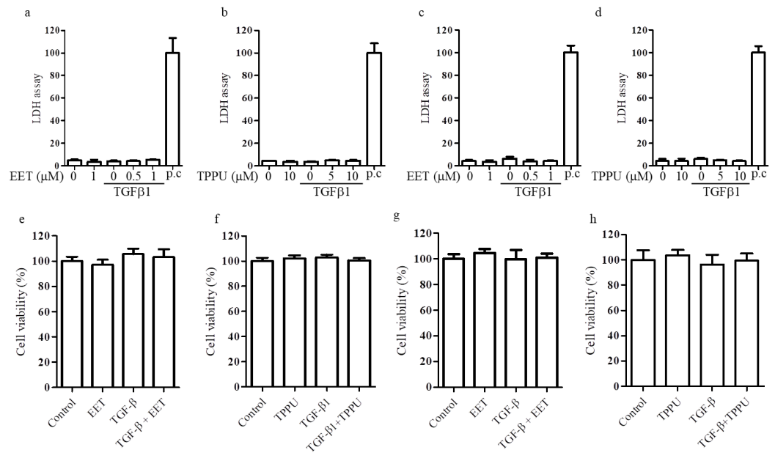


(a-b) MRC-5 cells were treated with TGF-β1 (5 ng/mL) for 24 h with/without 11,12-EET (a) or TPPU (b). (c-d) Beas-2B cells were treated with TGF-β1 (5 ng/mL) for 24 h with/without 11,12-EET (c) or TPPU (d). Cytotoxicity was then measured using lactate dehydrogenase (LDH) assay. Positive control (p.c), was acted as maximum LDH activity control which was condition medium from lysis buffer-treated cells. (e-f) MRC-5 cells were treated with TGF-β1 (5 ng/mL) for 24 h with/without 1 μL of 11,12-EET (e) or 10 μL of TPPU (f). (g-h) Beas-2B cells were treated with TGF-β1 (5 ng/mL) for 24 h with/without 1 μL of 11,12-EET (g) or 10 μL of TPPU (h). Cell viability was measured using MTT assay.

Supplementary Fig. S5. Reduced expression of E-cadherin in epithelial cells in response to 11,12-EET and TPPU.


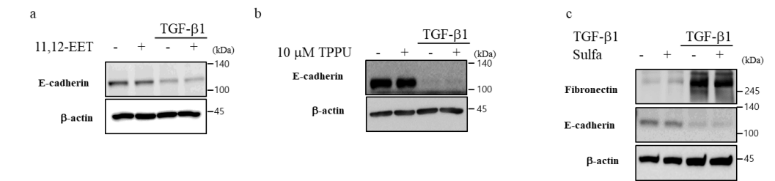


(a-b) Beas-2B cells were treated with 5 ng/mL of TGF-β1 for 24 h with/without 1 μM of 11,12-EET (a) or 10 μM of TPPU (b). (c) Beas-2B cells were treated with 5 ng/mL of TGF-β1 for 24 h with/without 20 μM of sulphaphenazole. Total cell extracts were prepared and subjected to western blotting using specific antibodies.

Supplementary Fig. S6. The effects of TPPU on the bleomycin-induced pulmonary fibrosis mouse model.


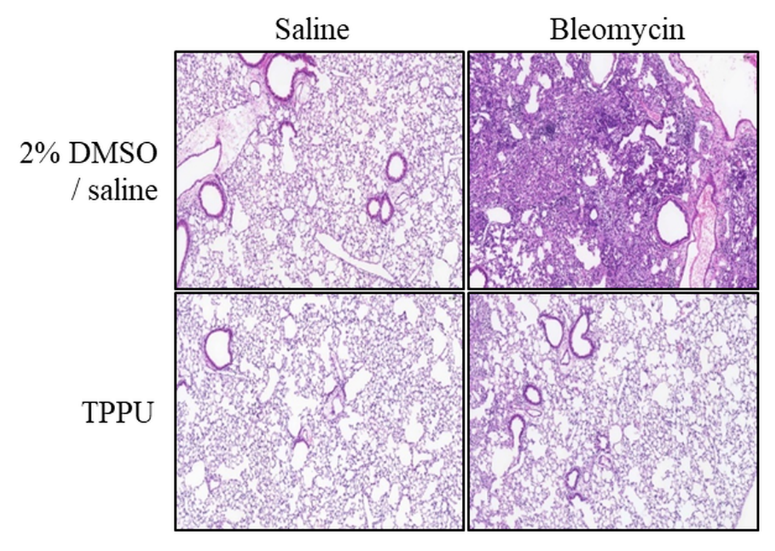


TPPU (0.25 mg/kg) was administered to mice 5 days/week for 3 weeks after bleomycin treatment (3 U/kg). Body weights were analysed for four groups of mice: control (n=3), bleomycin treatment (n=4), TPPU treatment (n=3), and bleomycin + TPPU treatment (n=6). Representative histological lung sections from each group stained with haematoxylin and eosin.
